# De‐Extinction at a Crossroads: Ecology, Ethics, and the Future of Conservation in the Biotech Age

**DOI:** 10.1111/ele.70217

**Published:** 2025-09-22

**Authors:** Bruno Paganeli, Mauro Galetti

**Affiliations:** ^1^ CBioClima‐Center for Research on Biodiversity Dynamics and Climate Change, Department of Biodiversity São Paulo State University (UNESP) Rio Claro São Paulo Brazil

**Keywords:** bioengineering, biological invasion, conservation ethics, De‐extinction, dire wolf, revival

## Abstract

The recent announcement of a genetically engineered organism resembling the extinct dire wolf (*Aenocyon dirus*) has reignited a media and academic debate over the ecological, ethical, and philosophical implications of de‐extinction. Although the revival was later clarified to be a modified grey wolf with a small fraction of the dire wolf DNA, the case illustrates how close biotechnology is to achieving a true de‐extinction. While proponents promote such techniques as innovative additional tools for conservation, this paper calls for a more critical examination of their broader implications. Drawing on insights from invasion ecology, rewilding, ethics, and governance, we argue that de‐extinction must not be guided by feasibility or commercial appeal alone. Instead, it requires a multidisciplinary framework to be thoroughly understood, responsibly guided, and—if deemed appropriate—accepted. As biotechnological innovations advance and may become widely used, they should be aligned with biodiversity conservation principles to avoid unintended ecological consequences.

## Graphical abstract

This paper critically examines the growing interest in de‐extinction, sparked by recent advances in biotechnology, such as the creation of an organism partially resembling the extinct dire wolf. While proponents see de‐extinction as a novel conservation tool, the authors argue that such efforts must be evaluated through a multidisciplinary lens that includes ecology, ethics, and governance. They emphasise that decisions should not be driven solely by technological capability or commercial interest, but must align with biodiversity conservation goals to avoid harmful ecological impacts.

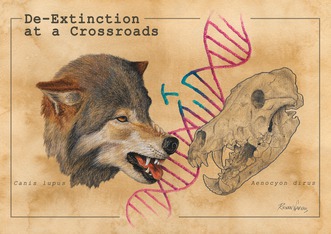

The idea of reviving extinct species has long been a controversial topic from conservation and biotechnology perspectives. Over the past decades, it has gained renewed attention, sparked by cases of species such as the woolly mammoth (*Mammuthus primigenius*) and the Pyrenean ibex ( *Capra pyrenaica pyrenaica* —Carlin et al. 2013). A potential definition of the de‐extinction process is that it should create an organism that is—or closely resembles—an extinct species (Martinelli et al. 2014). The dire wolf, *Aenocyon dirus*, formerly *Canis dirus* (Schmökel et al. 2023), is one of the currently extinct species that lived in the Americas (Reynolds et al. 2023) and parts of Eurasia (Lu et al. 2021) thousands of years ago. In early 2025, a genetically engineered organism resembling the dire wolf was announced as de‐extinct, though it was later clarified to be a modified grey wolf containing only a tiny proportion of dire wolf DNA (Lawton 2024). While not a true de‐extinction, this case illustrates how close current biotechnology is to achieving it, prompting discussion about its future direction. Supporters argue in the mass media that this strategy could expand the conservation toolbox. Here, we state that this claim requires thorough examination, especially when considering the ethical, evolutionary, and unforeseen ecological implications it can trigger.

Conservation efforts have traditionally focused on preventing ongoing extinctions, driven primarily by anthropogenic activities such as habitat loss, climate change, overexploitation of resources, and the spread of invasive, harmful species (Johnson et al. 2017). Nonetheless, should complex techniques like those applied to the dire wolf be refined and widely adopted to restore/conserve biodiversity? If so, should the focus be limited to species directly threatened by anthropogenic pressures or extended to those that went extinct due to natural causes (Schmökel et al. 2023)? If we broaden the scope, at what point does the time since extinction make revival ecologically unsuitable or even harmful? Should we consider the intrinsic value of species' existence, or prioritise those that contribute to ecosystem services and ultimately support human life? Furthermore, how can we ensure that species brought back to life are not only viable but also capable of thriving and developing/maintaining their well‐being? Given the likely limited number of individuals of ‘de‐extinct’ species, a major concern is the inevitable low genetic diversity of the founding population, which may compromise the long‐term viability of the species. Beyond the clear scientific motivation of turning theory into practice, should de‐extinction techniques be considered acceptable only when they lead to ecological outcomes that align with established conservation principles? Addressing these questions may not be straightforward, yet they stimulate important and timely scientific discussions.

The process of attempting to bring back extinct species is currently limited only by the availability of viable ancestral DNA and involves a series of complex and value‐laden decisions. Among them: which genome to reconstruct—the aimed extinct species itself, or a hybrid of a closely related living species? What ecological role, if any, should the resulting organism be expected to perform? What traits should be prioritised? Those points are far from neutral. Take, for instance, the trait of body size—a choice that carries a visual appeal. Colossal Laboratories, in its efforts to recreate certain traits of the extinct dire wolf, sequenced DNA and used CRISPR‐Cas9 techniques to edit approximately 20 key available genetic differences (loci) into 14 genes of modern grey wolves. By 6 months of age, the resulting puppies grow remarkably large. Males exceed 40 kg, making them 20% heavier than average grey wolves of the same age, while the female is about 10% larger than typical female grey wolves. This achievement marks a milestone in genetic engineering, demonstrating Colossal's ability to translate ancient DNA into living organisms that express targeted traits. Yet, the emphasis on size also has ecological implications. Large species (mainly endothermic ones) demand more energy and resources from the environment. This ecological cost, however, does not seem to be a concern for the research team. On the contrary, the company appears to aim for this trait, as shown by its plans to de‐extinct other large‐bodied species, such as the woolly mammoth and the giant moa (*Dinornis* spp.), a 3‐m‐tall, 250‐kg bird once native to New Zealand. What about other relatively smaller but symbolic species planned to be de‐extinct? For example, the dodo ( *Raphus cucullatus* ), extinct a few centuries ago, and the thylacine ( *Thylacinus cynocephalus* ), which went extinct less than a century ago, and whose ecological context has likely remained more stable. Are any of these cases an acceptable choice? This uncertainty reflects not a lack of scientific capacity, but rather the absence of clear regulatory frameworks and conservation‐oriented guidelines. As de‐extinction moves from theory to practice, and species choices appear to be guided predominantly by commercial and attention‐driven interests, this development raises fundamental questions: how do we define and value biodiversity that needs to be restored or conserved? These are not just conservation concerns—they demand urgent and coordinated reflection from ecological, ethical, and philosophical perspectives.

Even if these organisms are not intended for release, as the company once said, the possibility of a change in policy/opinion and—however unlikely—an accidental escape remains an important concern. While such events may appear improbable, past cases of species unintentionally escaping into the wild and disrupting local community dynamics (Koen et al. 2021) highlight the need for strict containment protocols and swift recapture procedures, assuming removal is ethically justified. Thus, acknowledging that the probability of escape may be low under strict oversight, the non‐zero risk still warrants consideration. In a case of escape, the situation may require integrating knowledge from multiple fields, especially invasion and rewilding ecology.

Biological invasion investigates species found outside their native range due to human‐mediated dispersal—a process recognised as a major driver of the current biodiversity crisis (Roy et al. 2024). Engineered species may not fit traditional definitions of nativeness. Yet, their uncertain ecological roles reinforce the need to define risk evaluation that centres on ecological performance and potential impacts—how a taxon might interact, spread, and adapt (IUCN 2020). Should an engineered species escape from closely monitored environments and go into the wild, the contemporary ecological forces might facilitate its expansion at the expense of coexisting species. For instance, the enemy release hypothesis (Keane and Crawley 2002) suggests that a species may spread more easily without natural enemies, a scenario especially relevant when considering engineered species. As a former apex and dominant predator (Lu et al. 2021), the dire wolf proxy could impose unintended and significant pressure on other organisms. These pressures include reducing prey and native species populations and disrupting ecological interactions shaped over evolutionary timescales (Nascimento et al. 2024). Moreover, even if vaccinated against certain diseases, the dire wolf proxy could facilitate the spread of other pathogens by acting as a novel or more efficient vector.

While rewilding strategies have been very beneficial in some cases, strategic interventions involving engineered species could produce a different—and possibly opposite—outcome. For instance, the rewilding of grey wolves in the northern hemisphere shows that the success of those activities is highly context‐dependent and might be even unpredictable (Smith and Peterson 2021). In heavily modified landscapes, such as much of Europe or urbanised North America, the ecological niche once occupied by the dire wolf may no longer exist. This likely mismatch between species demands and environmental characteristics could lead to failure in establishment (Paganeli et al. 2024), direct or territorial conflict with long‐standing species in the area, and unintended ecological outputs that can cascade to other trophic levels (Wilmers and Schmitz 2016). If the goal is not rewilding, then other facets of the discussion remain: how to define, quantify, and guarantee the well‐being of those species that will most likely miss ecological relationships from when they were a thriving species? Would such organisms fall under existing wildlife protection laws or require a new legal category specific to bioengineered life? Who would be responsible for their welfare, containment, or removal in case of accidental release and potential ecological harm? Such uncertainties might reach social‐ecological systems, including livestock management, wildlife governance and public perception. Public acceptance of large carnivore reintroductions has been shown to depend heavily on factors such as prior exposure, trusted information sources, and institutional credibility (Arbieu et al. 2019). These insights are crucial when considering the potential release—accidental or intentional—of engineered apex predators. Understanding and addressing these social dimensions is essential to support ecological responsibility and foster public trust.

As with ecological aspects, the rationale for de‐extinction—its benefits, risks and intended goals—is also formally lacking. For instance, it remains unclear whether the aim is to use those organisms for research or simply to showcase technological feasibility and bring attention from wealthy donors. One of the few points of agreement between supporters and critics is that we are witnessing a shift in how conservation is being discussed, including a potential paradigm shift founded on the perspective of biotechnology. Conservation biology has focused on protecting existing biodiversity, not reviving what has been lost. This distinction is crucial, as it influences how resources, attention and public trust are directed. There is a growing risk that conservation efforts may shift toward engineering ecosystems to serve more human‐centric interests. Thus, we argue that this discussion should be guided by ethicists, conservationists, ecologists, evolutionary biologists, bioengineers, economists, policymakers and supported through transparent communication with the public. Moreover, the emergence of neoecosystems—for example, urban areas hosting multiple non‐native species—illustrates that human‐driven species assemblages are not entirely new (Hobbs et al. 2006). De‐extinct species, therefore, may represent a continuous amplification of these trends. However, unlike past cases—such as biological invasions, where ecological risks were not promptly recognised or addressed—we now have the opportunity to guide these interventions from the outset, applying precautionary and multidisciplinary principles to prevent further biodiversity loss.

In parallel with ecological and other regulatory concerns, ethical considerations form a critical axis in evaluating the legitimacy of the likely upcoming de‐extinction realisation. Critics have long raised concerns about ‘playing God,’ questioning whether humans should interfere so deeply with evolutionary history. Animal welfare is another major concern. As we previously mentioned, what are the rights and well‐being of organisms created in laboratories, especially if they are destined for containment or experimental use? Furthermore, the financial cost of such projects is exorbitant. Estimates suggest that Colossal's efforts to engineer three dire wolf analogs exceeded $10 billion. Even acknowledging the autonomy of private funders, it is difficult to ignore the fact that such funding could have supported countless conservation initiatives aimed at protecting extant species. In a world where thousands of species are on the edge of extinction, the cost to de‐extinction species is not merely financial; it is profoundly ethical. Every dollar spent resurrecting the past is a dollar not spent to protect the present. This is especially true in megadiverse countries where funding is still very limited. If we have the means to engineer life, do we not also have the obligation to preserve it where it still exists? Philosophically, the idea that extinction can be reversed risks diminishing the perceived urgency of conservation. If extinction is seen as temporary or easily reversible, it may encourage complacency—or worse, justify ecologically unsustainable actions under the illusion that any damage can later be undone. This mindset of ‘extinction offsetting’ threatens to replace the moral and scientific foundations upon which conservation has long been built.

## 1 Conclusion

While scientific innovation progresses at an unprecedented rate, ethical and ecological considerations must remain central to conservation practices. If we can ever bring back to life a species with its full genetic material or closely resemble extinct species, we must also be prepared for an ecological role that may differ from what the evidence leads us to assume it once was, potentially driving biodiversity protection towards its decline. Ecological aspects are shaped not only by genetics but by complex interactions with environments that may no longer exist. Rewilding such species (intentionally or unintentionally) into modern ecosystems could disrupt existing ecological networks and pose new risks to biodiversity. To responsibly guide these efforts, a structured, multidisciplinary framework is essential. This includes thorough ecological risk assessments, ethical reviews akin to those in biomedical research, inclusive public engagement, and prioritisation of funding for protecting existing biodiversity.

## Author Contributions

Conceptualization: B.P. Supervision: M.G. Writing: B.P.

## Peer Review

The peer review history for this article is available at https://www.webofscience.com/api/gateway/wos/peer‐review/10.1111/ele.70217.

## Data Availability

This Viewpoint article does not use data or code.
